# Development of Predictive Modeling for Removal of Multispecies Biofilms of *Salmonella* Enteritidis, *Escherichia coli*, and *Campylobacter jejuni* from Poultry Slaughterhouse Surfaces

**DOI:** 10.3390/foods13111703

**Published:** 2024-05-29

**Authors:** Daiane Carvalho, Gabriela Zottis Chitolina, Daiane Elisa Wilsmann, Vivian Lucca, Brunna Dias de Emery, Karen Apellanis Borges, Thales Quedi Furian, Luciana Ruschel dos Santos, Hamilton Luiz de Souza Moraes, Vladimir Pinheiro do Nascimento

**Affiliations:** 1 Centro de Diagnóstico e Pesquisa em Patologia Aviária, Departamento de Medicina Animal, Faculdade de Veterinária, Universidade Federal do Rio Grande do Sul, Porto Alegre 91540-000, RS, Brazilthales.furian@ufrgs.br (T.Q.F.); moraeshls@gmail.com (H.L.d.S.M.); vladimir@ufrgs.br (V.P.d.N.); 2 Programa de Pós-Graduação em Bioexperimentação, Universidade de Passo Fundo, Passo Fundo 99052-900, RS, Brazil; luruschel@upf.br

**Keywords:** bacterial adhesion, *Campylobacter jejuni*, *Escherichia coli*, predictive modeling, *Salmonella* Enteritidis, poultry

## Abstract

*Salmonella* Enteritidis, *Escherichia coli*, and *Campylobacter jejuni* are among the most common foodborne pathogens worldwide, and poultry products are strongly associated with foodborne pathogen outbreaks. These pathogens are capable of producing biofilms on several surfaces used in the food processing industry, including polyethylene and stainless steel. However, studies on multi-species biofilms are rare. Therefore, this study aimed to develop predictive mathematical models to simulate the adhesion and removal of multispecies biofilms. All combinations of microorganisms resulted in biofilm formation with differences in bacterial counts. *E. coli* showed the greatest ability to adhere to both surfaces, followed by *S.* Enteritidis and *C. jejuni*. The incubation time and temperature did not influence adhesion. Biofilm removal was effective with citric acid and benzalkonium chloride but not with rhamnolipid. Among the generated models, 46 presented a significant coefficient of determination (R^2^), with the highest R^2^ being 0.88. These results provide support for the poultry industry in creating biofilm control and eradication programs to avoid the risk of contamination of poultry meat.

## 1. Introduction

Poultry products are widely consumed, and there is an increasing demand for these products worldwide. To meet demands, the concentration of broilers in the production environment is high, which increases the risk of disease spread. Some diseases can be transmitted to humans through the consumption of chicken meat [1]. *Salmonella* spp., *Escherichia coli*, and *Campylobacter* spp. are among the most common foodborne pathogens worldwide [2]. *Salmonella* Enteritidis and *Campylobacter jejuni* are responsible for foodborne illnesses in the USA and the European Union [3,4]. Poultry and poultry-derived products are the most common food products associated with salmonellosis and campylobacteriosis outbreaks [5]. In Brazil, *Salmonella* spp. and *E. coli* are among the most common bacteria associated with foodborne outbreaks [6]. Avian pathogenic *E. coli* strains may have zoonotic potential and can be transmitted from chicken meat to humans [7]. 

Surface adherence and biofilm formation are strategies adopted by several pathogens for survival outside the host [8]. The abilities of *S.* Enteritidis, *E. coli*, and *C. jejuni* to adhere to surfaces and produce biofilms at a broad range of temperatures and surfaces have been previously described [9,10,11,12,13,14,15]. These aggregated structures exhibit distinct community properties, including increased resistance to chemical disinfection [16]. After maturation, the biofilm may rupture, followed by detachment of the bacteria. The release of pathogenic microorganisms into the environment leads to product contamination, which is a major public health concern worldwide [17]. Thus, biofilms represent a significant risk factor for the spread of pathogens throughout the food chain [18]. 

There has been an increase in the number of biofilm-related studies, most of which are based on monospecies biofilm models. Biofilms in environmental habitats may contain multiple bacterial species [16,19,20]. Multispecies biofilms are more complex than monospecific biofilms. Thus, the extrapolation of the results obtained from in vitro monocultures to natural biofilms may be imprecise [20]. In addition, the presence of mixed species can favor the adherence of fastidious bacteria [19,21]. Previous studies that compared the adhesion capacity of mixed-species biofilms on polystyrene, stainless steel, and polyethylene surfaces found differences in the adhesion of *C. jejuni*, *S.* Enteritidis, and *E. coli*, regardless of the surface evaluated [22].

To ensure food safety, poultry slaughterhouses are routinely cleaned and disinfected to prevent bacterial adhesion. Owing to recently increased antimicrobial resistance, identifying alternative compounds of natural origin is essential. Previous studies demonstrated the potential of biosurfactants and organic acids to disrupt biofilm formation [9]. However, many factors affect the process of bacterial adhesion to surfaces, including bacterial and surface properties [22,23].

Recently, the number of studies on predictive modeling in microbiology has increased. It allows the prediction of the growth and activity of a microorganism in a given substrate over time using mathematical equations [24]. These predictions are based on the principle that the responses of microbial populations to environmental conditions are reproducible [25]. This approach is useful for hazard analysis and critical control point programs in food processing plants owing to the need to quantitatively deal with a range of variables that influence food safety. The effect of temperature on growth, inactivation, and survival of microorganisms was reported [26]. Despite its importance, few studies have elucidated the application of predictive modeling to better understand biofilms in the food industry.

This study aimed to develop predictive mathematical models to simulate the adhesion and inactivation of multispecies biofilms of *E. coli*, *S.* Enteritidis, and *C. jejuni* on stainless steel and polyethylene, considering temperature and microbial combinations as variables.

## 2. Materials and Methods

### 2.1. Bacterial Strains

One field strain of each species (*S.* Enteritidis, *E. coli*, and *C. jejuni*) was selected for this study. *S.* Enteritidis strains were isolated from a poultry farm environment, *E. coli* from a slaughterhouse surface, and *C. jejuni* from a refrigerated carcass. Strains were selected based on their biofilm-production ability, which was previously tested using a crystal violet assay at 4 °C (poultry slaughterhouses—handling environment temperature), 12 °C (Brazilian poultry slaughterhouses—maximum cutting room temperature), and 25 °C (average room temperature). These strains were selected based on their ability to produce biofilm at all temperatures on polyethylene and stainless steel [9]. *S.* Enteritidis and *E. coli* were stored at −80 °C in brain heart infusion (BHI) broth (Oxoid; Basingstoke, U.K.), supplemented with 15% glycerol (Synth; Diadema, Brazil). *C. jejuni* was stored at −80 °C in ultra-high-temperature-processed milk. Multispecies assays were performed with four combinations of microorganisms: (1) *S.* Enteritidis + *E. coli*, (2) *S.* Enteritidis + *C. jejuni*, (3) *E. coli* + *C. jejuni*, and (4) *S.* Enteritidis + *E. coli* + *C. jejuni*.

### 2.2. Inoculum Preparation

*S.* Enteritidis and *E. coli* were reactivated in BHI for 18 h at 37 °C and cultured on trypticase soy agar (Oxoid) for 24 h at 37 °C. One colony of each strain was suspended in 3 mL of trypticase soy broth (TSB) without glucose (BD Biosciences; Franklin Lakes, NJ, USA) for 18 h at 37 °C. McFarland standard No. 1 (Probac do Brasil, São Paulo, Brazil) was used as a reference to adjust the concentration of the bacterial suspension in TSB to 3 × 10^8^ colony-forming units (CFU)/mL. An SP-22 spectrophotometer (Biospectro; Curitiba, Brazil) was used to measure turbidity at 620 nm, which ranged from 0.224 to 0.300. *C. jejuni* was reactivated on blood agar (Kasvi; São José dos Pinhais, Brazil) supplemented with 5% defibrinated sheep blood (Newprov; Pinhais, Brazil), followed by incubation under microaerophilic conditions for 48 h at 42 °C.

### 2.3. Surface Preparation

Polyethylene and stainless steel (AISI 316) coupons with dimensions of 1 cm (width) × 1 cm (length) × 0.1 cm (thickness) were used in this study. The coupons were manually cleaned using a nonabrasive sponge, water, and neutral liquid detergent. The samples were then rinsed and immersed in distilled water for 24 h and 70% (*v*/*v*) ethyl alcohol for 24 h at room temperature. Finally, the samples were rinsed in distilled water. The coupons were sterilized at 121 °C for 30 min under pressure.

### 2.4. Compounds and Time of Contact

Three compounds were selected for this study: (1) biosurfactant rhamnolipid of *Pseudomonas aeruginosa* (R90; Agae Technologies, Corvallis, OR, USA) at 5% (*w*/*v*); (2) citric acid (C0759; Sigma-Aldrich, Darmstadt, Germany) at 10% (*w*/*v*); (3) disinfectant benzalkonium chloride (Êxodo Científica, Brazil) at 150 ppm. All the compounds were diluted in sterile distilled water. For the organic acids, stock solutions were prepared, and the pH was adjusted with HCl, when needed, to reach 3.1-4.8, based on its pKa values. The contact time was 10 min for all compounds. The concentrations of the compounds and contact times were selected based on a previous study [9].

### 2.5. Adhesion Test

To evaluate the biofilm formation, 3 mL aliquots were inoculated in 12-well flat-bottomed polystyrene plates (Kasvi). In combination with the two microorganisms, 1 mL of TSB without glucose and 1 mL of the bacterial suspension of each microorganism were added to each well. For combinations with three bacteria, 750 µL of TSB without glucose and 750 µL of the bacterial suspension of each microorganism were added per well. Stainless steel and polyethylene coupons were tested in triplicate and individually added to the wells. The plates were incubated at 4, 12, or 25 °C for 24 h. Individual plates were used for each combination of microorganisms, surface type, and temperature. After incubation, the coupons were individually removed using sterile tweezers and washed with 5 mL 0.1% buffered peptone water (BPW; Merck; Darmstadt, Germany) to remove the planktonic cells. The coupons were then placed in tubes containing 5 mL 0.1% BPW and rinsed with five glass beads (1 mm) on a vortex shaker (Kasvi) for 1 min to release the sessile cells.

After serially diluting the suspension using 0.85% saline solution, a bacterial count was performed for each coupon using the drop-plate technique [27] on xylose lysine deoxycholate (XLD) agar (Oxoid) for *S.* Enteritidis, eosin methylene blue (EMB) agar (Oxoid) for *E. coli*, and modified charcoal cefoperazone deoxycholate agar (mCCDA; Oxoid) plates with selective supplements (32 mg/L cefoperazone, 10 mg/L amphotericin B; SR0155; Oxoid) for *C. jejuni*. EMB and XLD agar plates were incubated at 37 °C for 24 h under aerobic conditions, and mCCDA plates were incubated under microaerophilic conditions at 42 °C for 48 h. To determine the microbiological count, colony forming units per square centimeter (CFU/cm^−2^), the surface area on both sides of the coupon, and the side area were considered:

(1) $$CFU/{cm}^{-2}=\left(\frac{VD}{VA}\right)\times AV\times \frac{D}{A}$$

where VD is the diluent volume used for rinsing (5 mL), VA is the aliquot volume used for plating (0.01 mL), AV is the average bacterial count on the plates (CFU), D is the dilution used for counting, and A is the coupon area (2 cm^2^). The results are expressed as log_10_ CFU/cm^−2^ [28,29].

### 2.6. Removal of Formed Biofilms

To evaluate removal of formed biofilms, the coupons were individually removed after incubation and were washed with 5 mL of 0.1% BPW to remove planktonic cells. The coupons were then placed in another plate containing 3 mL of each compound (rhamnolipid 5%, citric acid 10%, and benzalkonium chloride 150 ppm), in triplicate. The control group was incubated with sterile distilled water. Compounds were stored for 1 h at 4, 12, or 25 °C, according to the treatment, prior to their use. After inoculation, plates were maintained for 10 min at 4, 12, or 25 °C, according to the treatment. After incubation, the coupons were individually removed and placed in tubes containing 5 mL of 0.1% BPW with a neutralizer (polysorbate Tween 80 [Neon, São Paulo, Brazil], 2 g of soy lecithin [Stem, Porto Alegre, Brazil], and 2 g of sodium thiosulfate [Dynamic, Diadema, Brazil]) for 5 min at room temperature. The coupons were then rinsed with five glass beads (1 mm) on a vortex shaker for 1 min to release the sessile cells, and the bacterial count was performed as previously described.

### 2.7. Scanning Electron Microscopy (SEM)

Multispecies biofilm formation was examined using SEM. To perform the tests, the material was prepared according to a protocol developed by the Center for Microscopy and Microanalysis at the Federal University of Rio Grande do Sul (Porto Alegre, Brazil) [30].

The test was performed in sterile 12-well polystyrene microplates with stainless steel coupons. This surface was selected because previous studies with SEM analyses showed the increased irregularity of a stainless steel surface compared to that of polyethylene surfaces [22]. The standard inoculum (3 mL) at 10^8^ CFU·mL^−1^ was used and incubated on the microplate at 25 °C for 24 h. Subsequently, the coupons were washed with 5 mL of sterile distilled water to remove nonadherent cells. Then, the surfaces were immersed in a fixation solution (1.2 mL glutaraldehyde 25%, 5 mL 0.2 M phosphate buffer, and 3.8 mL distilled water) at 4 °C for seven days and washed thrice for 30 min each with 0.2 M phosphate buffer solution and distilled water (1:1), followed by dehydration in increasing acetone concentrations (30% for 10 min, 50% for 10 min, 70% for 10 min, 90% for 10 min, 90% for 20 min, 100% for 10 min, and 100% for 20 min) (Merck, Darmstadt, Germany). The coupons were dried using critical-point equipment (Balzers CPD030 BalTec; Pfäffikon, Switzerland) with liquid carbon dioxide as the transition fluid and overlaid with a 20 nm gold layer (SCD 050; BalTec). Analyses were performed using a JSM-6060 electron microscope (Jeol, Tokyo, Japan) operating at 10 kV and a Zeiss EVO MA 10 electron microscope (Zeiss; Oberkochen, Germany) operating at 8 kV.

### 2.8. Development of Predictive Mathematical Models for Multispecies Biofilms

Mathematical linear regression models were developed based on the following variables: (1) microorganism combination (*S.* Enteritidis + *E. coli*; *S.* Enteritidis + *C. jejuni*; *E. coli* + *C. jejuni*; *S.* Enteritidis + *E. coli* + *C. jejuni*), (2) temperature (4, 12, or 25 °C), (3) surface (stainless steel or polyethylene), (4) incubation time (4, 12, or 24 h), and (5) treatment (control, rhamnolipid 5%, citric acid 10%, and benzalkonium chloride 150 ppm). The variable to be predicted was the number of cells adhered to the surfaces for each microorganism, and the results are expressed in CFU/cm^−2^. The fit of the models was obtained by analyzing the coefficients of determination. All equations and coefficients of determination were obtained using GraphPad Prism software 9.0.1 (Prism, San Diego, CA, USA), with a significance level of 5%.

### 2.9. Statistical Analyses

All statistical analyses were performed using GraphPad software 9.0.1, with a significance level of 5%. The results are expressed as CFU/cm^−2^ and transformed into log_10_ CFU/cm^−2^. One-way ANOVA, followed by Bonferroni’s post hoc comparison test, was performed to compare adhesion at different temperatures and surfaces in multispecies biofilms, and the differences among biofilm removal treatments.

## 3. Results

### 3.1. Adhesion

Biofilm formation was observed in all combinations of microorganisms (*S.* Enteritidis + *E. coli*, *S.* Enteritidis + *C. jejuni*, *E. coli* + *C. jejuni*, *S.* Enteritidis + *E. coli* + *C. jejuni*), temperatures (4, 12, and 25 °C), and surfaces (polyethylene and stainless steel). The recovery rate of microorganisms ranged from 10^5^ CFU/cm^−2^ (5 log_10_ CFU/cm^−2^) to 10^6^ CFU/cm^−2^ (6 log_10_ CFU/cm^−2^) for *S.* Enteritidis and *E. coli* and was approximately 10^4^ CFU/cm^−2^ (4 log_10_ CFU/cm^−2^) for *C. jejuni*, regardless of the conditions tested. A comparison of the adhesion of *S.* Enteritidis, *E. coli*, and *C. jejuni* to stainless steel and polyethylene, considering different microbial combinations, is presented in Table 1. *E. coli* showed the greatest ability to adhere to both surfaces, followed by *S.* Enteritidis and *C. jejuni* (*p* < 0.05), regardless of the microbial combination or temperature. There were no significant differences (*p* > 0.05) in the adhesion of microorganisms among the incubation times for the same microbial combination, temperature, or surface.

**Figure 1:**
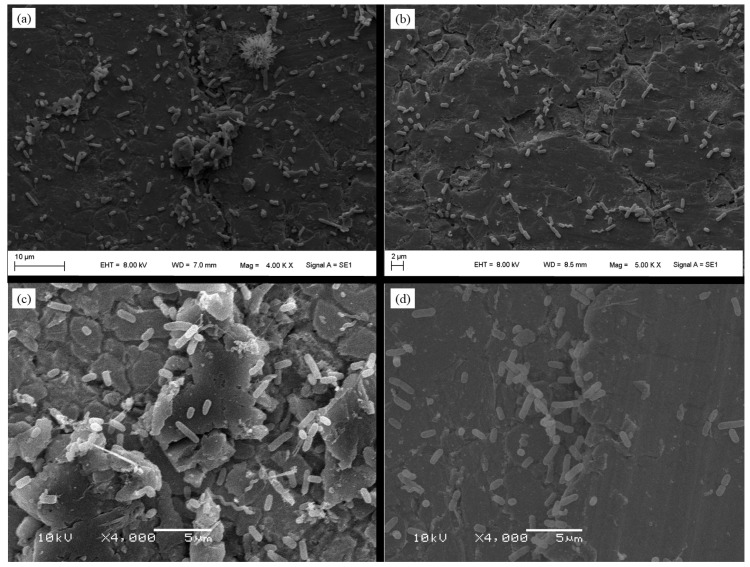
Multispecies biofilm formed on stainless steel at 25 °C after 24 h of incubation: (**a**) *Salmonella* Enteritidis + *Escherichia coli*, (**b**) *Salmonella* Enteritidis + *Campylobacter jejuni*, (**c**) *Escherichia coli* + *Campylobacter jejuni*, and (**d**) *Salmonella* Enteritidis + *Escherichia coli* + *Campylobacter jejuni*.

The SEM images demonstrate the adhesion of *S.* Enteritidis, *E. coli*, and *C. jejuni* on stainless steel for all combinations of microorganisms (Figure 1). The images also demonstrate increased irregularity on stainless steel.

### 3.2. Biofilm Removal

Bacterial counts after the removal treatments for each combination of microorganisms, temperature, and surface area are described in Table 2, Table 3, Table 4 and Table 5. In all combinations, treatment with rhamnolipids showed similar results (*p* > 0.05) as the control group, regardless of the microorganism, temperature, or surface. In most cases, citric acid completely eliminated the biofilm of *S.* Enteritidis, *E. coli*, and *C. jejuni*. Benzalkonium chloride also caused significant (*p* < 0.05) reductions in bacterial counts after treatment with all microorganisms under the majority of conditions evaluated. The effect of the surface on biofilm removal was more evident for *S.* Enteritidis, where polyethylene produced better results than that with stainless steel. The influence of temperature on bacterial removal varied among the experiments.

### 3.3. Predictive Mathematical Models

Simple linear regression models were generated based on the plate counts of the multispecies microbial combinations. Models were generated for each microorganism, considering different microbial combinations, surfaces, temperatures, and treatments. All models were valid for a range between 4 and 24 h, allowing the prediction of the number of bacterial cells that adhered to polyethylene and stainless steel, as well as the number of viable cells remaining after treatment with antimicrobials. The response variable is given in log_10_ CFU/cm^−2^. The data were obtained from three observations for each condition. It was not possible to generate a model for treatments with counts equal to zero in all three observations.

In total, 160 models were built, 46 of which presented a statistically significant coefficient of determination (R^2^), explaining the adhesion or the effect of the treatments on the multispecies biofilms under the evaluated conditions. The microbial combination that provided the most valid mathematical models was *S.* Enteritidis + *E. coli* + *C. jejuni*, with 15 models, followed by *S.* Enteritidis + *E. coli*, with 13 models. The combination of *S.* Enteritidis + *C. jejuni* and *E. coli* + *C. jejuni* yielded nine valid models. Among the valid models, 19 referred to the adhesion of microorganisms to surfaces (control), 18 to treatment with rhamnolipid, 8 to benzalkonium chloride, and 1 to citric acid. The highest R^2^ was 0.88 for *S.* Enteritidis + *C. jejuni* for the treatment with rhamnolipid at 25 °C, and the lowest was 0.41 for *S.* Enteritidis + *E. coli* + *C. jejuni* for the treatment with benzalkonium chloride at 25 °C. These models are shown in the Supplementary Material.

## 4. Discussion

Multispecies biofilms are more common and represent the most important lifestyle types in the natural environment [31,32]. Thus, studies evaluating multispecies biofilms are required to better understand interspecies interactions. The species that constitute a multispecies biofilm directly influence the interactions between the microorganisms. Therefore, in this study, we selected four combinations of *S.* Enteritidis, *E. coli,* and *C. jejuni*. These pathogens are often linked to foodborne disease outbreaks worldwide [2,4] and are known for their ability to form biofilms [9,10,11,12,13,14,15].

In this study, all combinations of microorganisms resulted in biofilm formation, with differences in bacterial counts. Previous studies with monospecies biofilms demonstrated that *C. jejuni* adhesion was significantly lower than *S.* Enteritidis and *E. coli* adhesion, regardless of the surface evaluated [22]. Similarly, in this study, *E. coli* showed the greatest ability to adhere to both surfaces, followed by *S.* Enteritidis. *C. jejuni* presented less bacterial adhesion, regardless of the microbial combination or temperature. Interactions between microorganisms may affect bacterial adhesion because they influence the development, shape, and functional dynamics of biofilms [16,32,33]. However, it is likely that the low adhesion of *C. jejuni* is related to its intrinsic characteristics or it can enter a “viable-but-not-cultivable” state, which makes its recovery difficult [34]. Thus, it is possible that the interactions among these three species do not necessarily result in synergistic or antagonistic effects. Further studies are required to understand the nature of these interactions.

Rhamnolipids are effective antimicrobial compounds against monospecies biofilms of *S.* Enteritidis, *E. coli,* and *C. jejuni* [9]. However, in this study, rhamnolipids did not significantly reduce bacterial counts, regardless of the conditions evaluated. It is likely that the antimicrobial action of biosurfactants was reduced when tested in mixed cultures [35]. Several studies have evaluated the antimicrobial and antibiofilm activities of rhamnolipids after long periods (>2 h) of contact [35,36,37]. However, prolonged exposure times do not simulate ideal conditions for use in food processing plants. Therefore, the contact times of 5 and 10 min, which were tested in the present study, are closer to those used in the actual routines in the food industry. Citric acid and benzalkonium chloride significantly reduced the bacterial counts of all microorganisms under most of the evaluated conditions, which is consistent with the results of previous studies [38]. It was observed that, in some cases, there was greater biofilm removal on polyethylene than on stainless steel. However, to a large extent, this difference may have been the result of the adhesion effect on this surface, which was weaker than that on steel in most situations, similar to previously described findings [22]. These results demonstrate that temperature did not influence the adhesion of different microorganisms, in contrast to the results observed for monospecies biofilms [11,13,39,40].

The use of predictive models for studying biofilms has gained interest over time. Studies on predictive models for multispecies biofilms are scarce. To the best of our knowledge, there have been no reports of models that combine *S.* Enteritidis, *E. coli*, and *C. jejuni* under conditions that simulate microbial adhesion and inactivation in the food-processing industry to date. Valderrama [41] developed regression models to demonstrate differences in biofilm formation between different strains of *L. monocytogenes*. The results demonstrate the importance of understanding the environmental factors affecting biofilm production by this pathogen, allowing differentiation of adhesion between strains and justifying the prevalence of specific lineages in specific habitats. Bernardes [42] developed models to evaluate the adhesion of *Bacillus cereus* to stainless steel depending on time and temperature. Temperature had a greater influence on the adhesion of this pathogen to stainless steel, with good adjustment of the models. Chmielewski and Frank [43] proposed heat inactivation models for mono- (*L. monocytogenes*) and multispecies (*L. monocytogenes* + *Pseudomonas* spp. + *Pantoea agglomerans*) biofilms and demonstrated that the models could be an important tool for adapting hot-water sanitization processes on rubber surfaces.

Several models can be used to study biofilms, including flow balance analysis, statistical inference, growth kinetics, and genetic modeling [44]. A statistical inference model was used in this study. This model assumes that a single species is rarely found within a biofilm [44].

The accuracy of the models can be calculated and compared using the analysis of coefficient of multiple determination (R^2^) and analysis of variance. R^2^ represents how much of the variation in a result can be predicted by the model, and it is an indicator of how efficiently the model fits the data. R^2^ values near “1” indicate higher predictive capacity [45,46]. For this study, R^2^ >50% was considered satisfactory because these models are based on multispecies biofilms, where numerous variables influence the adhesion and inactivation of microorganisms. Among the constructed models, 20 presented an R^2^ > 0.60 (Supplementary Material). The *S.* Enteritidis prediction model for polyethylene and the *E. coli* prediction model for stainless steel with rhamnolipid 5% presented R^2^ values > 80% (Supplementary Material) and would be selected as the best fit.

Linear regression models must consist of a continuous dependent variable and independent variables, which can be continuous, binary, or categorical [47]. All these assumptions were met in this study to develop the model. However, the presence of categorical (surface, temperature, and microbial combination) and numerical (biofilm formation time) variables did not allow the generation of multivariate analysis models. Notably, the majority of the models generated were related to adhesion to surfaces or treatment with rhamnolipids. In both cases, the bacterial counts differed from zero. Although these models did not present significant R^2^ values, they increased the number of models generated. In addition, treatment with citric acid and benzalkonium chloride resulted in the total removal of bacterial cells, making it impossible to build representative models.

In conclusion, all combinations of microorganisms resulted in biofilm formation with differences in bacterial counts. The incubation time and temperature did not influence adhesion. Biofilm removal was effective with citric acid and benzalkonium chloride. Among the generated models, those for *S.* Enteritidis and *E. coli* with rhamnolipid 5% on polyethylene and stainless steel, respectively, presented the best fit and should be selected for future studies. These results provide support for the poultry industry in creating biofilm control and eradication programs to avoid the risk of contamination of poultry meat.

## Figures and Tables

**Table 1:** Bacterial count (log_10_ CFU/cm^−2^) for comparison of the adhesion of *Salmonella* Enteritidis, *Escherichia coli*, and *Campylobacter jejuni* on stainless steel and polyethylene, regardless of temperature.

	*Salmonella* Enteritidis + *Escherichia coli*		*Salmonella* Enteritidis + *Campylobacter jejuni*		*Escherichia coli* + *Campylobacter jejuni*		*Salmonella* Enteritidis + *Escherichia coli* + *Campylobacter jejuni*	
	Stainless Steel	Polyethylene	Stainless Steel	Polyethylene	Stainless Steel	Polyethylene	Stainless Steel	Polyethylene
*Salmonella* Enteritidis	6.26 ^a^	5.43 ^a^	5.92 ^a^	5.62 ^a^	-	-	6.14 ^a^	5.78 ^a^
*Escherichia coli*	6.66 ^b^	6.15 ^b^	-	-	6.40 ^a^	6.09 ^a^	6.35 ^a^	6.13 ^b^
*Campylobacter jejuni*	-	-	4.29 ^b^	4.23 ^b^	4.32 ^b^	4.40 ^b^	4.40 ^b^	4.57 ^c^

Lowercase letters in the same column indicate a significant difference among microorganisms (Bonferroni test; *p* < 0.05).

**Table 2:** Bacterial count (log_10_ CFU/cm^−2^) of multispecies *Salmonella* Enteritidis and *Escherichia coli* biofilms on stainless steel and polyethylene at 4, 12, and 25 °C after treatment (rhamnolipid 5%, citric acid 10%, and benzalkonium chloride 150 ppm).

Treatment	Temperature (°C)	*Salmonella* Enteritidis		*Escherichia coli*	
		Polyethylene	Stainless Steel	Polyethylene	Stainless Steel
Control	4	5.30 ± 0.17 ^aA^	6.12 ± 0.20 ^bA^	5.65 ± 0.6 ^aA^	6.69 ± 0.22 ^bA^
	12	5.26 ± 0.22 ^aA^	6.19 ± 0.32 ^bA^	6.25 ± 0.35 ^aA^	6.71 ± 0.28 ^bA^
	25	5.72 ± 0.07 ^aB^	6.46 ± 0.19 ^bB^	6.54 ± 0.35 ^aB^	6.57 ± 0.39 ^aA^
Rhamnolipid	4	5.07 ± 0.22 ^aA^	5.76 ± 0.18 ^bA^	5.41 ± 0.67 ^aA^	6.18 ± 1.52 ^bA^
	12	5.26 ± 0.31 ^aA^	6.07 ± 0.17 ^bB^	6.16 ± 0.30 ^aB^	6.23 ± 1.67 ^aA^
	25	5.33 ± 0.31 ^aA^	6.26 ± 0.14 ^bB^	6.33 ± 0.34 ^aB^	6.36 ± 1.55 ^aA^
Citric Acid	4	**0** **^aA^**	**0.44 ± 0.93 ^aA^**	**0 ^aA^**	**0 ^aA^**
	12	**0 ^aA^**	**0 ^aA^**	**0 ^aA^**	**0 ^aA^**
	25	**0.29 ± 0.87 ^aA^**	**0.66 ± 1.32 ^aA^**	**0 ^aA^**	**1.32 ± 1.59 ^bB^**
Benzalkonium Chloride	4	3.10 ± 1.79 ^aA^	**3.85 ± 0.73 ^aA^**	3.23 ± 2.05 ^aA^	**3.37 ± 1.52 ^aA^**
	12	**1.59 ± 1.52 ^aA^**	**2.81 ± 1.67 ^bA^**	**1.03 ± 1.60 ^aB^**	**1.09 ± 1.67 ^aB^**
	25	**0.75 ± 1.49 ^aB^**	4.20 ± 1.75 ^bA^	**0.58 ± 1.15 ^aB^**	**0.78 ± 1.55 ^aB^**

Legend: Different lowercase letters on the same line indicate a statistical difference (*p* < 0.05) between surfaces for the same microorganism, treatment, and temperature. Different uppercase letters in the same column indicate a statistical difference (*p* < 0.05) among temperatures for the same microorganism, treatment, and surface. Bold values indicate a statistical difference (*p* < 0.05) between the treatment and the control under the same conditions (microorganism, surface, and temperature).

**Table 3:** Bacterial count (log_10_ CFU/cm^−2^) of multispecies *Salmonella* Enteritidis and *Campylobacter jejuni* biofilms on stainless steel and polyethylene at 4, 12, and 25 °C after treatment (rhamnolipid 5%, citric acid 10%, and benzalkonium chloride 150 ppm).

Treatment	Temperature (°C)	*Salmonella* Enteritidis		*Campylobacter jejuni*	
		Polyethylene	Stainless Steel	Polyethylene	Stainless Steel
Control	4	4.97 ± 0.23 ^aA^	6.07 ± 0.39 ^bA^	4.17 ± 0.32 ^aA^	4.43 ± 0.14 ^aA^
	12	5.96 ± 0.64 ^aB^	5.76 ± 0.25 ^aA^	4.11 ± 0.28 ^aA^	4.28 ± 0.24 ^aA^
	25	5.93 ± 0.68 ^aB^	5.93 ± 0.41 ^aA^	4.41 ± 0.33 ^aA^	4.15 ± 0.24 ^aA^
Rhamnolipid	4	4.13 ± 1.58 ^aA^	5.48 ± 0.26 ^bA^	3.97 ± 0.12 ^aA^	3.96 ± 0.15 ^aA^
	12	5.74 ± 0.82 ^aB^	5.47 ± 0.23 ^aA^	4.38 ± 0.40 ^aA^	4.22 ± 0.12 ^aA^
	25	6.16 ± 0.63 ^aB^	5.77 ± 0.39 ^aA^	4.01 ± 0.26 ^aA^	3.86 ± 1.48 ^aA^
Citric Acid	4	**0 ^aA^**	**0 ^aA^**	**0 ^aA^**	**0 ^aA^**
	12	**0 ^aA^**	**0 ^aA^**	**0 ^aA^**	**0 ^aA^**
	25	**0 ^aA^**	**0 ^aA^**	**0 ^aA^**	**0.74 ± 1.30 ^aA^**
Benzalkonium Chloride	4	2.79 ± 1.66 ^aA^	**3.78 ± 2.19 ^aA^**	**0 ^aA^**	**0.34 ± 1.50 ^aA^**
	12	**0.97 ± 1.47 ^aB^**	**1.86 ± 1.82 ^aA^**	**0 ^aA^**	**0.45 ± 1.03 ^aA^**
	25	**0.38 ± 1.13 ^aB^**	**1.38 ± 1.64 ^aB^**	**0.40 ± 1.19 ^aA^**	**0 ^aA^**

Legend: Different lowercase letters on the same line indicate a statistical difference (*p* < 0.05) between surfaces for the same microorganism, treatment, and temperature. Different uppercase letters in the same column indicate a statistical difference (*p* < 0.05) among temperatures for the same microorganism, treatment, and surface. Bold values indicate a statistical difference (*p* < 0.05) between the treatment and the control under the same conditions (microorganism, surface, and temperature).

**Table 4:** Bacterial count (log_10_ CFU/cm^−2^) of multispecies *Escherichia coli* and *Campylobacter jejuni* biofilms on stainless steel and polyethylene at 4, 12, and 25 °C after treatment (rhamnolipid 5%, citric acid 10%, and benzalkonium chloride 150 ppm).

Treatment	Temperature (°C)	*Escherichia coli*		*Campylobacter jejuni*	
		Polyethylene	Stainless Steel	Polyethylene	Stainless Steel
Control	4	5.73 ± 0.19 ^aA^	6.39 ± 0.09 ^bA^	4.34 ± 0.23 ^aA^	4.23 ± 0.21 ^aA^
	12	6.31 ± 0.29 ^aB^	6.39 ± 0.13 ^aA^	4.31 ± 0.22 ^aA^	4.08 ± 0.43 ^aA^
	25	6.23 ± 0.22 ^aB^	6.43 ± 0.42 ^aA^	4.55 ± 0.20 ^aA^	4.66 ± 0.13 ^aA^
Rhamnolipid	4	5.45 ± 0.38 ^aA^	6.20 ± 0.16 ^bA^	3.89 ± 0.35 ^aA^	3.84 ± 0.33 ^aA^
	12	6.17 ± 0.37 ^aB^	6.26 ± 0.07 ^aA^	4.19 ± 0.39 ^aA^	3.73 ± 0.55 ^aA^
	25	6.12 ± 0.23 ^aB^	6.35 ± 0.23 ^aA^	4.40 ± 0.26 ^aA^	3.62 ± 1.40 ^aA^
Citric Acid	4	**0 ^aA^**	**0 ^aA^**	**0 ^aA^**	**0 ^aA^**
	12	**0 ^aA^**	**0 ^aA^**	**0 ^aA^**	**0 ^aA^**
	25	**0.44 ± 1.31 ^aA^**	**0.41 ± 1.23 ^aA^**	**0 ^aA^**	**0.73 ± 1.45 ^aA^**
Benzalkonium Chloride	4	2.66 ± 1.58 ^aA^	3.59 ± 1.60 ^aA^	**0.68 ± 1.36 ^aA^**	**0 ^aA^**
	12	**0.44 ± 1.31 ^aB^**	**2.05 ± 2.00 ^bA^**	**0.41 ± 1.22 ^aA^**	**0 ^aA^**
	25	**0 ^aB^**	**1.34 ± 1.62 ^bB^**	**0 ^aA^**	**0 ^aA^**

Legend: Different lowercase letters on the same line indicate statistical difference (*p* < 0.05) between surfaces for the same microorganism, treatment, and temperature. Different uppercase letters on the same column indicate statistical difference (*p* < 0.05) among temperatures for the same microorganism, treatment, and surface. Bold values indicate statistical difference (*p* < 0.05) between the treatment and the control at the same conditions (microorganism, surface, and temperature).

**Table 5:** Bacterial count (log_10_ CFU/cm^−2^) of multispecies *Salmonella* Enteritidis, *Escherichia coli*, and *Campylobacter jejuni* biofilms on stainless steel and polyethylene at 4, 12, and 25 °C after treatment (rhamnolipid 5%, citric acid 10%, and benzalkonium chloride 150 ppm).

Treatment	Temperature (°C)	*Salmonella* Enteritidis		*Escherichia coli*		*Campylobacter jejuni*	
		Polyethylene	Stainless Steel	Polyethylene	Stainless Steel	Polyethylene	Stainless Steel
Control	4	5.51 ± 0.27 ^aA^	6.33 ± 0.38 ^bA^	5.91 ± 0.17 ^aA^	6.40 ± 0.39 ^bA^	4.47 ± 0.17 ^aA^	4.28 ± 0.64 ^aA^
	12	5.88 ± 0.32 ^aB^	5.96 ± 0.13 ^aA^	6.37 ± 0.20 ^aB^	6.56 ± 0.19 ^aA^	4.53 ± 0.24 ^aB^	4.34 ± 0.35 ^bA^
	25	5.94 ± 0.42 ^aB^	6.13 ± 0.52 ^aA^	6.12 ± 0.07 ^aB^	6.10 ± 0.22 ^aB^	4.70 ± 0.09 ^aB^	4.58 ± 0.35 ^aA^
Rhamnolipid	4	5.25 ± 0.39 ^aA^	6.02 ± 0.42 ^bA^	5.54 ± 0.22	6.21 ± 0.38	4.19 ± 0.33 ^aA^	4.17 ± 2.05 ^aA^
	12	5.45 ± 0.22 ^aAB^	5.62 ± 0.36 ^aA^	5.93 ± 0.31	6.35 ± 0.24	4.44 ± 0.37 ^aB^	4.15 ± 1.60 ^aA^
	25	5.89 ± 0.50 ^aB^	5.75 ± 0.22 ^aA^	5.70 ± 0.31	5.60 ± 0.42	4.48 ± 0.12 ^aB^	3.99 ± 1.15 ^bA^
Citric Acid	4	**0 ^aA^**	**0 ^aA^**	**0 ^aA^**	**0 ^aA^**	**0 ^aA^**	**0 ^aA^**
	12	**0 ^aA^**	**0 ^aA^**	**0 ^aA^**	**0 ^aA^**	**0 ^aA^**	**0 ^aA^**
	25	**0.32 ± 0.97 ^aA^**	**0. 39 ± 0.97 ^aA^**	**0.29 ± 0.87 ^aA^**	**0 ^aA^**	**0 ^aA^**	**0 ^aA^**
Benzalkonium Chloride	4	**2.75 ± 1.58 ^aA^**	**3.96 ± 1.67 ^aA^**	**2.89 ± 1.80 ^aA^**	**3.19 ± 1.32 ^aA^**	**0 ^aA^**	**0 ^aA^**
	12	**0.71 ± 1.44 ^aB^**	**3.54 ± 1.40 ^bA^**	**0.36 ± 1.52 ^aB^**	**1.54 ± 1.47 ^aA^**	**0 ^aA^**	**0 ^aA^**
	25	**1.11 ± 1.69 ^aB^**	**2.19 ± 1.70 ^aA^**	**0.29 ± 1.50 ^aB^**	**2.07 ± 1.97 ^bA^**	**0 ^aA^**	**0 ^aA^**

Legend: Different lowercase letters on the same line indicate a statistical difference (*p* < 0.05) between surfaces for the same microorganism, treatment, and temperature. Different uppercase letters in the same column indicate a statistical difference (*p* < 0.05) among temperatures for the same microorganism, treatment, and surface. Bold values indicate a statistical difference (*p* < 0.05) between the treatment and the control under the same conditions (microorganism, surface, and temperature).

## Data Availability

The original contributions presented in the study are included in the article/Supplementary Material, further inquiries can be directed to the corresponding author.

## Supplementary Materials

The following supporting information can be downloaded at: https://www.mdpi.com/article/10.3390/foods13111703/s1.
