# Supplementary material for: Development of Predictive Modeling for Removal of Multispecies Biofilms of Salmonella Enteritidis, Escherichia coli, and Campylobacter jejuni from Poultry Slaughterhouse Surfaces
Source: Foods. 2024 May 29;13(11):1703. doi: 10.3390/foods13111703 (PMC11172265; doi:10.3390/foods13111703)
Supplement: Supplementary file 1 [file foods-13-01703-s001.zip › foods-3025719-supplementary.pdf]

SUPPLEMENTARY MATERIAL

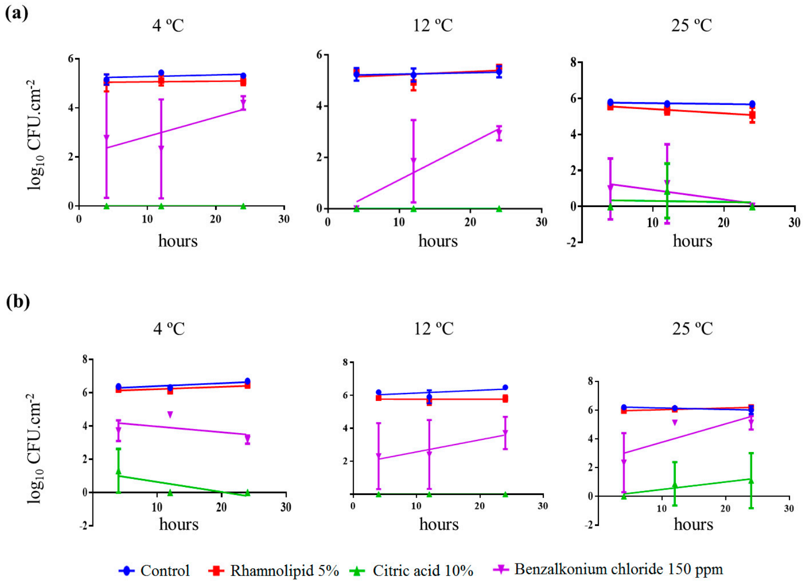

Regression equations (R^2) – polyethylene

Temperature (°C)	Control	Rhamnolipid 5%	Citric acid 10%	Benzalkonium chloride 150 ppm
4	Y = 0.006517 × X + 5.214 (0.10)	Y = 0.002491 × X + 5.038 (0.009)	NA	Y = 0.07848 × X + 2.053 (0.14)
12	Y = 0.005455 × X + 5.191 (0.048)	Y = 0.01242 × X + 5.095 (0.12)	NA	**Y = 0.1425 × X - 0.3047 (0.66)**
25	Y = -0.004709 × X + 5.783 (0.29)	Y = -0.02379 × X + 5.648 (0.43)	Y = -0.005745 × *X + 0.3677 (0.003)	Y = -0.05316 × X + 1.455 (0.09)

Regression equations (R^2) – stainless steel

Temperature (°C)	Control	Rhamnolipid 5%	Citric acid 10%	Benzalkonium chloride 150 ppm
4	**Y = 0.01737 × X + 6.225 (0.58)**	**Y = 0.01403 × X + 6.076 (0.52)**	Y = -0.06032 × X + 1.241 (0.32)	Y = -0.03448 × X + 4.315 (0.16)
12	Y = 0.01711 × X + 5.966 (0.21)	Y = -0.0001920 × X + 5.768 (0.19)	NA	Y = 0.07335 × X + 1.835 (0.14)
25	Y = -0.009832 × X + 6.253 (0.21)	**Y = 0.01144 × X + 5.922 (0.50)**	Y = 0.05248 × X - 0.03988 (0.12)	Y = 0.1279 × X + 2.497 (0.40)

Figure S1. *Salmonella* Enteritidis prediction models in polyethylene **(a)** and stainless steel **(b)** at 4, 12, and 25 °C for biofilm formed by *Salmonella* Enteritidis and *Escherichia coli*.

Legend:

Bold equations indicate significant R^2 ($P < 0.05$).

NA: Not available (treatments with no bacterial growth in three observations).

$R^2 > 60\%$ (yellow)

$R^2 > 80\%$ (purple)

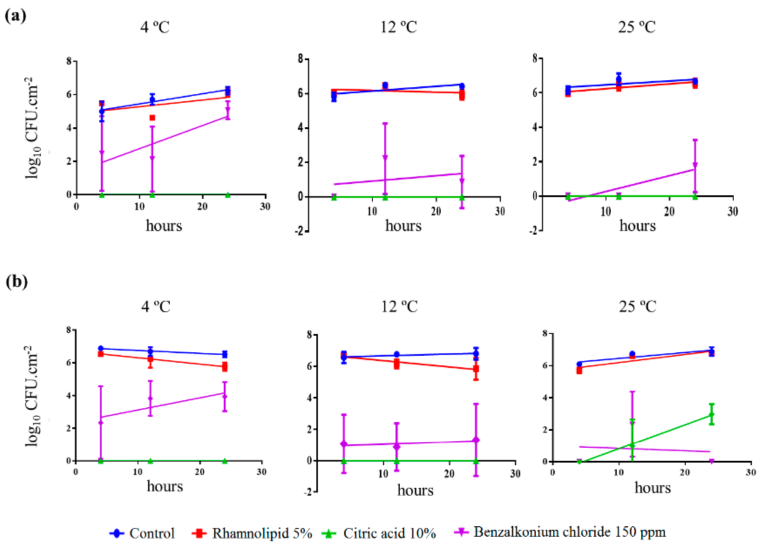

Regression equations (R^2) – polyethylene

Temperature (°C)	Control	Rhamnolipid 5%	Citric acid 10%	Benzalkonium chloride 150 ppm
4	**Y = 0.06064 × X + 4.842 (0.67)**	Y = 0.04003 × X + 4.878 (0.26)	NA	Y = 0.1385 × X + 1.381 (0.34)
12	**Y = 0.02735 × X + 5.885 (0.46)**	Y = -0.009794 × X + 6.289 (0.082)	NA	Y = 0.03133 × X + 0.6145 (0.029)
25	Y = 0.02257 × X + 6.241 (0.30)	**Y = 0.02826 × X + 5.959 (0.52)**	NA	**Y = 0.09192 × X - 0.6435 (0.48)**

Regression equations (R^2) – stainless steel

Temperature (°C)	Control	Rhamnolipid 5%	Citric acid 10%	Benzalkonium chloride 150 ppm
4	**Y = -0.01788*X + 6.933 (0.47)**	**Y = -0.03878*X + 6.697 (0.56)**	NA	Y = 0.07398*X + 2.389 (0.17)
12	Y = 0.01128*X + 6.559 (0.12)	Y = -0.03993*X + 6.761 (0.44)	NA	Y = 0.01465*X + 0.8962 (0.005)
25	**Y = 0.03661*X + 6.085 (0.66)**	**Y = 0.05054*X + 5.687 (0.75)**	**Y = 0.1504*X - 0.6878 (0.67)**	Y = -0.01545*X + 0.9889 (0.007)

Figure S2. *Escherichia coli* prediction models in polyethylene **(a)** and stainless steel **(b)** at 4, 12, and 25 °C for biofilm formed by *Salmonella* Enteritidis and *Escherichia coli*.
Legend:
Bold equations indicate significant R^2 ($P < 0.05$).
NA: Not available (treatments with no bacterial growth in three observations).
$R^2 > 60\%$
$R^2 > 80\%$

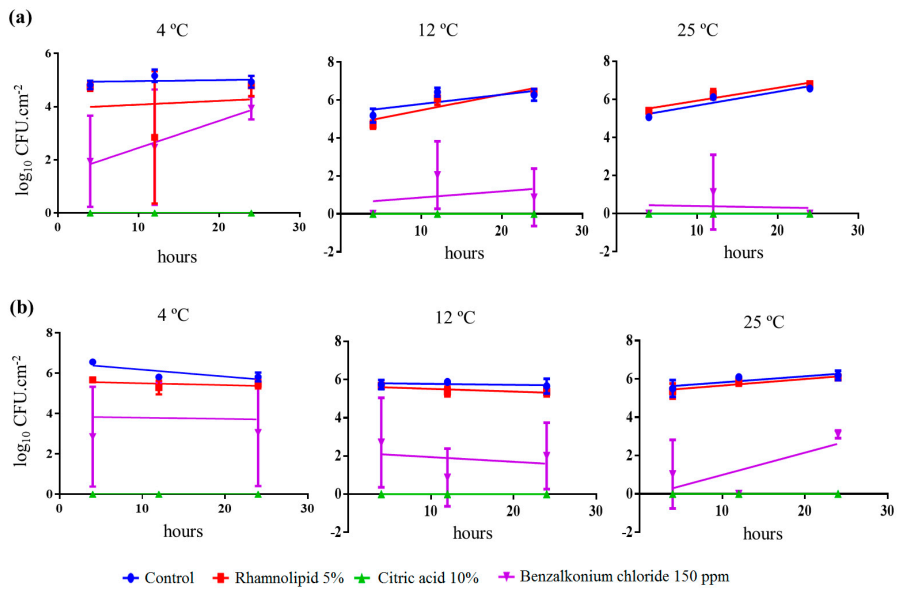

Regression equations (R^2) – polyethylene

Temperature (°C)	Control	Rhamnolipid 5%	Citric acid 10%	Benzalkonium chloride 150 ppm
4	Y = 0.003958 × X + 4.922 (0.021)	Y = 0.01426 × X + 3.935 (0.00)	NP	Y = 0.1017 × X + 1.434 (0.28)
12	**Y = 0.04926 × X + 5.303 (0.44)**	**Y = 0.08257 × X + 4.644 (0.76)**	NP	Y = 0.03249 × X + 0.5404 (0.03)
25	**Y = 0.07260 × X + 4.958 (0.86)**	**Y = 0.06784 × X + 5.257 (0.88)**	NP	Y = -0.007452 × X + 0.4769 (0.00)

Regression equations (R^2) – stainless steel

Temperature (°C)	Control	Rhamnolipid 5%	Citric acid 10%	Benzalkonium chloride 150 ppm
4	**Y = -0.03415 × X + 6.523 (0.57)**	Y = -0.009066 × X + 5.597 (0.088)	NA	Y = -0.005862 × X + 3.857 (0.000)
12	Y = -0.005070 × X + 5.828 (0.03)	Y = -0.01427 × X + 5.660 (0.29)	NA	Y = -0.02462 × X + 2.189 (0.01)
25	**Y = 0.03192 × X + 5.504 (0.44)**	**Y = 0.03411 × X + 5.317 (0.58)**	NA	Y = 0.1163 × X - 0.1686 (0.38)

Figure S3. *Salmonella* Enteritidis prediction models in polyethylene **(a)** and stainless steel **(b)** at 4, 12, and 25 °C for biofilm formed by *Salmonella* Enteritidis and *Campylobacter jejuni*.
Legend:
Bold equations indicate significant R^2 ($P < 0.05$).
NA: Not available (treatments with no bacterial growth in three observations).
$R^2 > 60\%$ (orange)
$R^2 > 80\%$ (purple)

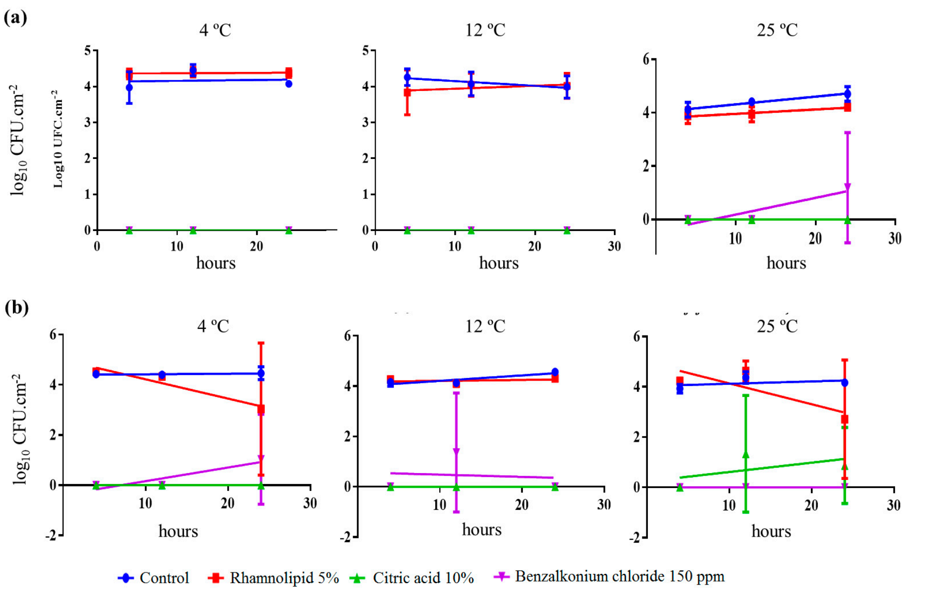

Regression equations (R^2) – polyethylene

Temperature (°C)	Control	Rhamnolipid 5%	Citric acid 10%	Benzalkonium chloride 150 ppm
4	Y = 0.002360 × X + 4.137 (0.00)	Y = 0.0009015 × X + 4.364 (0.00)	NA	NA
12	Y = -0.01318 × X + 4.285 (0.16)	Y = 0.008264 × X + 3.858 (0.03)	NA	NA
25	**Y = 0.02910 × X + 4.022 (0.59)**	Y = 0.01648 × X + 3.793 (0.31)	NA	Y = 0.06270 × X - 0.4389 (0.21)

Regression equations (R^2) – stainless steel

Temperature (°C)	Control	Rhamnolipid 5%	Citric acid 10%	Benzalkonium chloride 150 ppm
4	Y = 0.002058 × X + 4.398 (0.01)	Y = -0.07680 × X + 4.987 (0.20)	NA	Y = 0.05433 × X - 0.3803 (0.21)
12	**Y = 0.02162 × X + 3.994 (0.61)**	Y = 0.003483 × X + 4.175 (0.06)	NA	Y = -0.008984 × X + 0.5750 (0.00)
25	Y = 0.009302 × X + 4.029 (0.00)	Y = -0.08293 × X + 4.965 (0.23)	NA	Y = 0.03715 × X + 0.2422 (0.04)

Figure S4. *Campylobacter jejuni* prediction models in polyethylene **(a)** and stainless steel **(b)** at 4, 12, and 25 °C for biofilm formed by *Salmonella* Enteritidis and *Campylobacter jejuni*.

Legend:

Bold equations indicate significant R^2 ($P < 0.05$).

NA: Not available (treatments with no bacterial growth in three observations).

$R^2 > 60\%$ (yellow)

$R^2 > 80\%$ (purple)

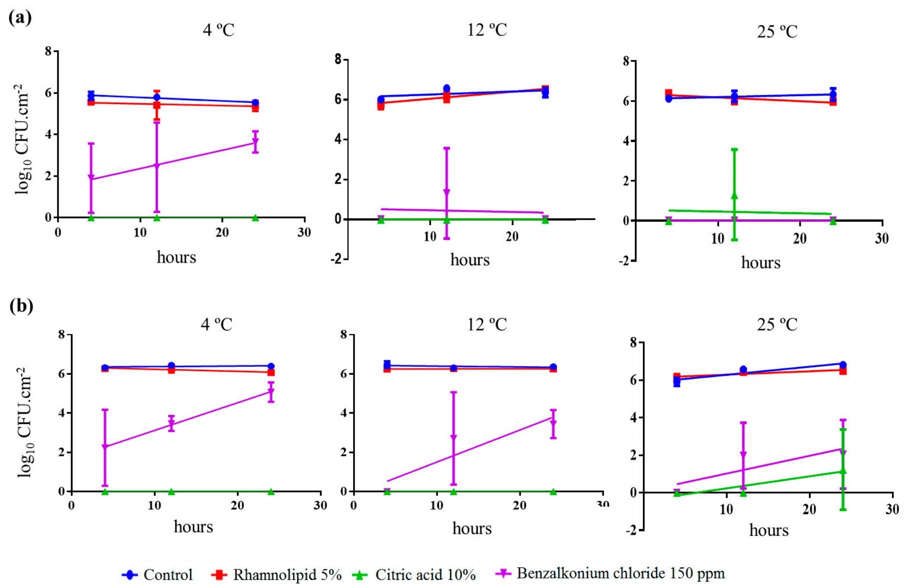

Regression equations (R^2) – polyethylene

Temperature (°C)	Control	Rhamnolipid 5%	Citric acid 10%	Benzalkonium chloride 150 ppm
4	**Y = -0.01686 × X + 5.956 (0.58)**	Y = -0.008447 × X + 5.564 (0.03)	NA	Y = 0.08822 × X + 1.485 (0.23)
12	Y = 0.01498 × X + 6.114 (0.20)	**Y = 0.03544 × X + 5.698 (0.69)**	NA	Y = -0.008598 × X + 0.5503 (0.00)
25	Y = 0.009660 × X + 6.104 (0.14)	**Y = -0.01883 × X + 6.373 (0.50)**	NA	Y = -0.008598 × X + 0.5503 (0.00)

Regression equations (R^2) – stainless steel

Temperature (°C)	Control	Rhamnolipid 5%	Citric acid 10%	Benzalkonium chloride 150 ppm
4	Y = 0.003125 × X + 6.345 (0.09)	Y = -0.01055 × X + 6.346 (0.33)	NA	Y = 0.1412 × X + 1.713 (0.59)
12	Y = -0.004386 × X + 6.445 (0.08)	Y = 0.0004068 × X + 6.255 (0.00)	NA	Y = 0.1636 × X - 0.1276 (0.51)
25	**Y = 0.04319 × X + 5.854 (0.79)**	**Y = 0.01797 × X + 6.114 (0.45)**	Y = 0.06409 × X - 0.4029 (0.20)	Y = 0.09474 × X + 0.07912 (0.26)

Figure S5. *Escherichia coli* prediction models in polyethylene **(a)** and stainless steel **(b)** at 4, 12, and 25 °C for biofilm formed by *Escherichia coli* and *Campylobacter jejuni*.

Legend:

Bold equations indicate significant R^2 ($P < 0.05$).

NA: Not available (treatments with no bacterial growth in three observations).

$R^2 > 60\%$

$R^2 > 80\%$

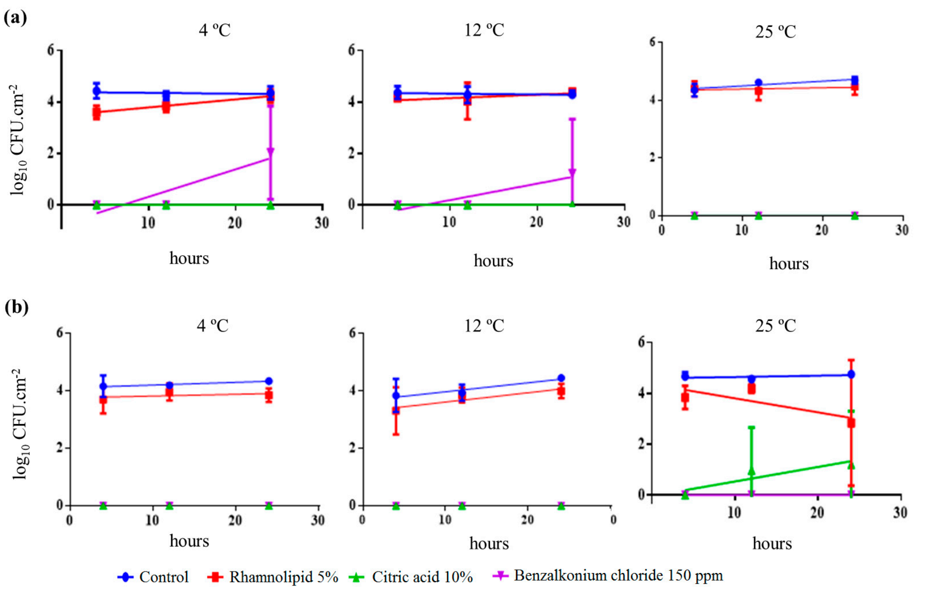

Regression equations (R^2) – polyethylene

Temperature (°C)	Control	Rhamnolipid 5%	Citric acid 10%	Benzalkonium chloride 150 ppm
4	Y = -0.003073 × X + 4.383 (0.01)	**Y = 0.03122 × X + 3.475 (0.61)**	NA	**Y = 0.1067 × X - 0.7472 (0.46)**
12	Y = -0.003362 × X + 4.359 (0.01)	Y = 0.01328 × X + 4.017 (0.08)	NA	Y = 0.06423 × X - 0.4496 (0.21)
25	**Y = 0.01615 × X + 4.340 (0.49)**	Y = 0.004600 × X + 4.341 (0.02)	NA	NA

Regression equations (R^2) – stainless steel

Temperature (°C)	Control	Rhamnolipid 5%	Citric acid 10%	Benzalkonium chloride 150 ppm
4	Y = 0.009460 × X + 4.107 (0.14)	Y = 0.006155 × X + 3.758 (0.02)	NA	NP
12	Y = 0.03128 × X + 3.665 (0.40)	Y = 0.03273 × X + 3.290 (0.11)	NA	NP
25	Y = 0.005225 × X + 4.587 (0.11)	Y = -0.05510* × + 4.355 (0.11)	NA	Y = 0.05710 × X – 0.03460 (0.11)

Figure S6. *Campylobacter jejuni* prediction models in polyethylene **(a)** and stainless steel **(b)** at 4, 12, and 25 °C for biofilm formed by *Escherichia coli* and *Campylobacter jejuni*.
Legend:
Bold equations indicate significant R^2 ($P < 0.05$).
NA: Not available (treatments with no bacterial growth in three observations).
$R^2 > 60\%$
$R^2 > 80\%$

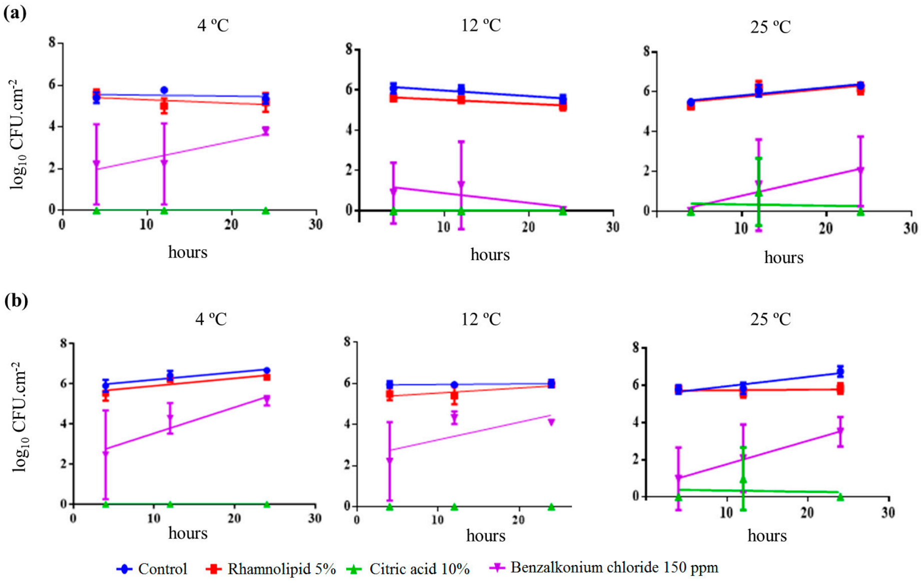

Regression equations (R^2) – polyethylene

Temperature (°C)	Control	Rhamnolipid 5%	Citric acid 10%	Benzalkonium chloride 150 ppm
4	Y = -0.005137 × X + 5.580 (0.02)	Y = -0.01663 × X + 5.474 (0.13)	NA	Y = 0.08394 × X + 1.635 (0.21)
12	**Y = -0.02791 × X + 6.257 (0.58)**	**Y = -0.01957 × X + 5.708 (0.60)**	NA	Y = -0.04847 × X + 1.356 (0.08)
25	**Y = 0.04055 × X + 5.405 (0.71)**	**Y = 0.03997 × X + 5.354 (0.48)**	Y = -0.006405 × X + 0.4099 (0.00)	Y = 0.09689 × X - 0.1829 (0.25)

Regression equations (R^2) – stainless steel

Temperature (°C)	Control	Rhamnolipid 5%	Citric acid 10%	Benzalkonium chloride 150 ppm
4	**Y = 0.03682 × X + 5.835 (0.68)**	**Y = 0.03773 × X + 5.517 (0.60)**	NA	**Y = 0.1297 × X + 2.233 (0.45)**
12	Y = 0.002902 × X + 5.925 (0.03)	Y = 0.02444 × X + 5.294 (0.03)	NA	Y = 0.08549 × X + 2.405 (0.28)
25	Y = 0.05044 × X + 5.456 (0.70)	Y = 0.003038 × X + 5.714 (0.01)	Y = -0.006405 × X + 0.4099 (0.00)	**Y = 0.1260 × X + 0.5065 (0.41)**

Figure S7. *Salmonella* Enteritidis prediction models in polyethylene **(a)** and stainless steel **(b)** at 4, 12, and 25 °C for biofilm formed by *Salmonella* Enteritidis, *Escherichia coli*, and *Campylobacter jejuni*.
Legend:
Bold equations indicate significant R^2 ($P < 0.05$).
NA: Not available (treatments with no bacterial growth in three observations).
$R^2 > 60\%$ (yellow)
$R^2 > 80\%$ (purple)

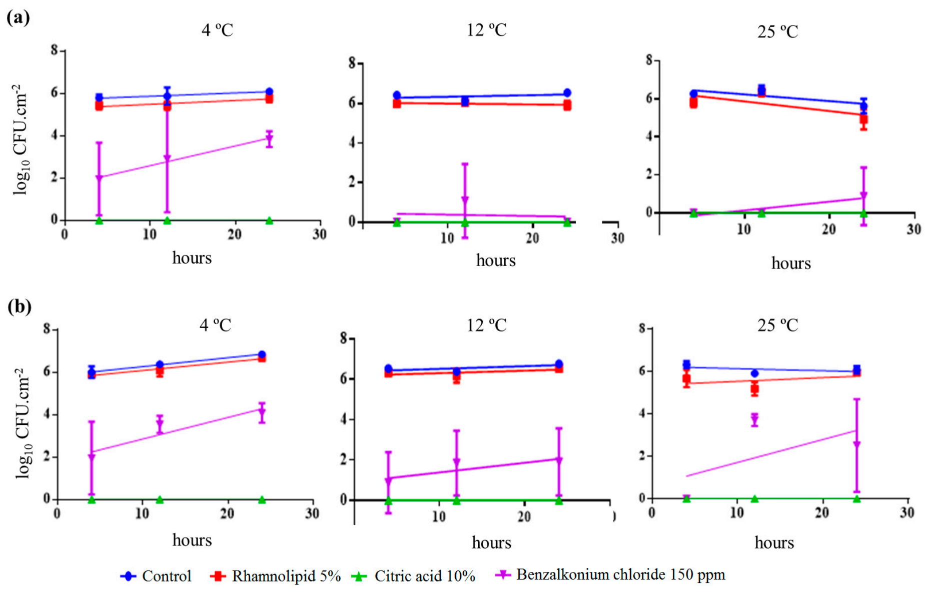

Regression equations (R^2) – polyethylene

Temperature (ºC)	Control	Rhamnolipid 5%	Citric acid 10%	Benzalkonium chloride 150 ppm
4	Y = 0.01553 × X + 5.706 (0.27)	Y = 0.01807 × X + 5.299 (0.40)	NA	Y = 0.09304 × X + 1.654 (0.21)
12	Y = 0.008136 × X + 6.260 (0.09)	Y = -0.004544 × X + 6.044 (0.09)	NA	Y = -0.007065 × X + 0.4522 (0.00)
25	**Y = -0.03540 × X + 6.593 (0.46)**	Y = -0.05075 × X + 6.376 (0.39)	NA	Y = 0.04596 × X – 0.3217 (0.21)

Regression equations (R^2) – stainless steel

Temperature (ºC)	Control	Rhamnolipid 5%	Citric acid 10%	Benzalkonium chloride 150 ppm
4	**Y = 0.04188 × X + 5.843 (0.85)**	**Y = 0.03937 × X + 5.686 (0.81)**	NA	**Y = 0.1016 × X + 1.835 (0.44)**
12	Y = 0.01334 × X + 6.383 (0.38)	Y = 0.01223 × X + 6.183 (0.19)	NA	Y = 0.04793 × X + 0.9029 (0.08)
25	Y = -0.009918 × X + 6.236 (0.15)	Y = 0.01745 × X + 5.363 (0.13)	NA	Y = 0.1079 × X + 0.6369 (0.22)

Figure S8. *Escherichia coli* prediction models in polyethylene **(a)** and stainless steel **(b)** at 4, 12, and 25 °C for biofilm formed by *Salmonella* Enteritidis, *Escherichia coli*, and *Campylobacter jejuni*.
Legend:
Bold equations indicate significant R^2 ($P < 0.05$).
NA: Not available (treatments with no bacterial growth in three observations).
$R^2 > 60\%$
$R^2 > 80\%$

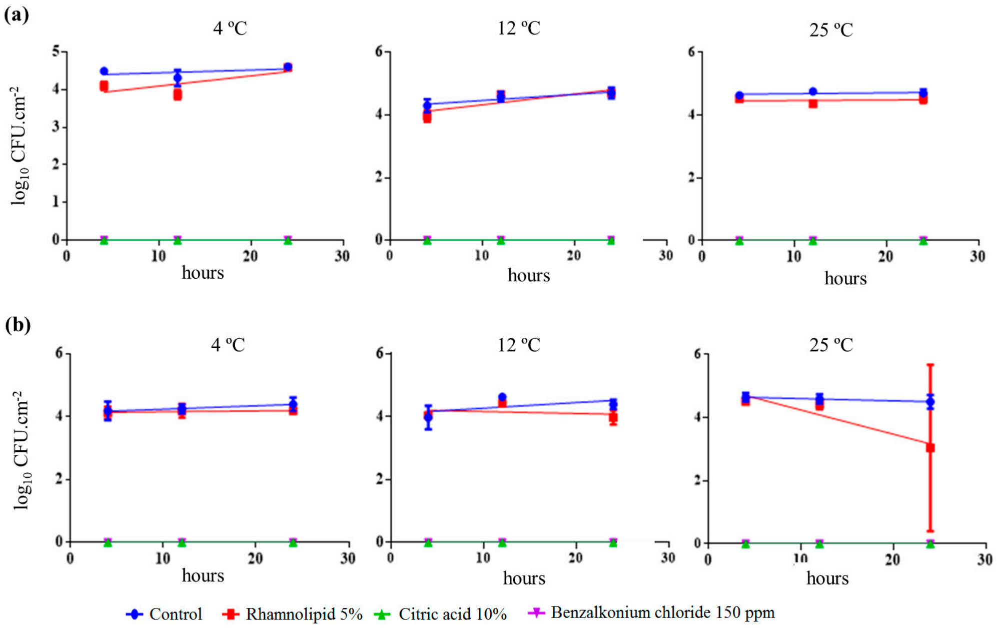

Regression equations (R^2) – polyethylene

Temperature (°C)	Control	Rhamnolipid 5%	Citric acid 10%	Benzalkonium chloride 150 ppm
**4**	Y = 0.007309 × X + 4.375 (0.14)	**Y = 0.02725 × X + 3.824 (0.50)**	NA	NA
**12**	**Y = 0.01931 × X + 4.270 (0.50)**	**Y = 0.03402 × X + 3.987 (0.62)**	NA	NA
**25**	Y = 0.002631 × X + 4.666 (0.06)	Y = 0.001651 × X + 4.455 (0.01)	NA	NA

Regression equations (R^2) – stainless steel

Temperature (°C)	Control	Rhamnolipid 5%	Citric acid 10%	Benzalkonium chloride 150 ppm
**4**	Y = 0.01068 × X + 4.135 (0.18)	Y = 0.003107 × X + 4.127 (0.02)	NA	NA
**12**	Y = 0.01777 × X + 4.102 (0.19)	Y = -0.006111 × X + 4.235 (0.04)	NA	NA
**25**	Y = -0.006982 × X + 4.676 (0.14)	Y = -0.07659 × X + 5.012 (0.19)	NA	NA

Figure S9. *Campylobacter jejuni* prediction models in polyethylene **(a)** and stainless steel **(b)** at 4, 12, and 25 °C for biofilm formed by *Salmonella* Enteritidis, *Escherichia coli*, and *Campylobacter jejuni*.
Legend:
Bold equations indicate significant R^2 ($P < 0.05$).
NA: Not available (treatments with no bacterial growth in three observations).
$R^2 > 60\%$
$R^2 > 80\%$
